# Report of a Case of Fracture of the Basis Cranii, Terminating in Inflammation of the Brain, and Fatal Effusion; with the Results of a Juridical Examination of the Body

**Published:** 1817-07

**Authors:** Henry Ward, Shirley Palmer

**Affiliations:** Member of the Royal College of Surgeons in London


*Report of a Case of Fracture of the Basis Cranii, terminating in Inflammation of the Brain, and fatal Effusion; with the Results of a Juridical Examination of the Body.* By HENRY WARD, Esq. Member of the Royal College of Surgeons in London; and SHIRLEY PALMER, M. D.

### Mr. WARD'S REPORT.

BETWEEN eight and nine o'clock, on Saturday night, the 16th of November last, a healthy middle-aged man, on his return from Atherstone to his residence at Hartshill, distant about three miles, was found lying upon the road, wounded and insensible. He was immediately conveyed to his house, a few hundred yards from the spot, and put to bed. From ignorance of the extent of the injuries which he had sustained, or other cause with which I am not acquainted, the accident of the man was not publicly talked of; and two days had nearly elapsed ere, at the instance of a neighbouring gentleman, surgical aid was applied for.

I first saw the man on Monday the 18th, at noon. He was then labouring under complicated symptoms of compression and concussion of the brain, in a severe form. He could not distinctly articulate, and the pupil of the eye was somewhat dilated and nearly insensible. There was a small wound of the scalp in the occipital region; but neither fracture nor depression could be detected on the introduction of a probe. Upon several parts of the body were traces of violence hereafter to be described. *Two pounds of blood were drawn from the arm, and a large dose of hydrargyri submurias, and of magnesiæ sulphas in infusion of senna, given to evacuate the bowels.*

19th. His condition was much the same. *Blood was again drawn to the amount of a pound, and the purgatives continued.*

20th. No change. *One pound of blood again taken. Remedies continued.*

21st. No change either in the symptoms or treatment, but the bleeding not repeated.

22d. The feverish symptoms were declining. My patient was more sensible to external impressions, and apparently much relieved, but still unable to give me any account of the manner in which his wounds had been inflicted. *Solution of acetate of ammonia* with *tincture of digitalis was now substituted for the purgatives; and a dose of extract of henbane administered at bed-time.*

23d. State little altered. *Medicines continued.*

24th. Sunk again into a state of utter insensibility. *Bleeding repeated to one pound: medicines continued.*

25th. Much worse. Symptoms of delirium had shewn themselves. *Twenty ounces of blood were drawn from the external jugular vein, and the purgative plan was resumed.* Towards evening, the condition of my patient appeared almost desperate; and, as a last resource, it was determined, in a consultation with some other surgeons, to apply the trephine to the occiput. The symptoms rendered it probable that an effusion of blood or pus had taken place upon the membranes in that region; by the evacuation of which the man might be rescued from impending destruction. On separating the scalp, the various structures composing it, were seen in a very bloody and inflamed state; but, as far as the incision extended, no fracture or depression of the bone was observed. When the circle of cranium had been removed, the dura mater was discovered in a highly inflamed, livid, and almost disorganized condition. A small quantity of bloody sanious fluid issued from a rupture in the centre of the exposed portion. No relief followed the operation, during which the patient had not evinced any sign of sensibility; and nothing unusual had occurred. The wound was dressed in the ordinary manner.

26th. Symptoms still unabated. *Twelve ounces of blood were drawn from the temporal artery.* That night the patient died.

Having described the symptoms and treatment of this mysterious case, I shall now resign to Dr. Palmer, the task of detailing the results of a dissection, conducted with great care and minute attention to every circumstance which could throw light on the obscure subject of our investigation.

### Dr. Palmer's Report.

On Thursday afternoon, November 28th, I repaired, with Mr. Ward, to the dwelling of the deceased, in order to inspect the body previously to the arrival of the coroner. Three other medical gentlemen, Mr. Perkins of Measham, Mr. Bucknill of Nuneaton, and Mr. Clay, late of Atherstone, had the kindness to meet us there. Fully impressed with the responsibility which future events might attach to our conduct on this occasion, and anxious to enforce by example the precepts and admonitions which I had recently delivered on the juridical inspection of bodies through the medium of this Journal, I entered upon the dissection, in the determination that nothing should escape my notice, calculated, in the slightest degree, to resolve my own doubts, or to influence the decisions of the jury, respecting the mode in which this unfortunate man met with his fatal injuries.

The following notes were sketched by myself during the examination, and subsequently read and acceded to by all the professional gentlemen present.

### *Notes of the Dissection of the Body of E. B. aged 45, performed about forty-four Hours after his Decease.*

*External view.* Marks of severe injury of the scalp existed in the occipital region. There was a small triangular funnel-shaped wound, penetrating to the bone, and situated somewhat above, and to the right of, the occipital tubercle. It appeared to have been inflicted by an angular body. An extravasation of blood was seen beneath the membrane of the lips, and traces of a violent concussion of the loins, at the junction of the last lumbar vertebra with the sacrum. The cuticle was abraded from each shin to the extent of more than an inch, at the distance of about six inches from the inferior boundary of the patella. The body seemed to have been that of a healthy and vigorous man. Not the slightest vestige of previous disease was externally visible. The axillæ, the groins, the interior of the mouth, the nose, and all the accessible cavities, were explored with the most scrupulous attention.

*Abdomen* and *Thorax.* The various organs contained in these cavities were, upon the whole, remarkably healthy. A considerable degree of redness, differing widely in appearance from common inflammation, was exhibited, however, by the mucous membrane of the stomach, near its cardiac orifice, and a deep pink blush was diffused over the internal coat of the aorta, from the origin of that vessel to its curve, but without valvular disease or other morbid alteration. There existed not even the slightest adhesions of the pleuræ. The heart was in a perfectly sound and natural state.

*Cranium.* No adhesion had taken place between the scalp separated in the operation, and the bone. The dura mater, seen through the trephine orifice, had a bloody, deeply inflamed, and almost gangrenous appearance. Upon removing the calvaria, both that membrane and the pia mater were found highly inflamed in the vicinity of the wound; at some points, preternaturally adhering together; in others, containing between them effused blood. The lateral ventricles, and the third and fourth also, were completely distended by a turbid bloody fluid. The plexus choroides displayed evident traces of increased action; as did the brain generally, and particularly its investing membranes. The organ having been removed, a very considerable fracture was discovered, extending more than four inches in length, and implicating both tables of the bone. It commenced in the vicinity of the external wound, to the right of the occipital tubercle, took an oblique direction downward and to the left, crossed the perpendicular spine of the bone about its middle, and terminated in the foramen magnum, just behind the left occipital condyle. The separation of the margins of the fracture was such, that the blade of a strong scalpel might with facility be introduced, and moved to and fro in it. On the anterior portion of the left hemisphere, at the distance of about one inch and a half above the supraciliary ridge, a thin layer of effused blood, as large as a shilling, lay between the dura and pia mater; and the former membrane bore evident marks of severe concussion there; but the bone and integuments, covering that portion of the dura mater, had no unnatural appearance.

After a deliberate reconsideration of all the facts and circumstances which this dissection had unfolded, a report,* founded on the preceding notes, was conjointly

* In all cases of juridical examination of bodies, I would recommend the physician or surgeon conducting it, to draw up a report, in which the facts presenting themselves on dissection, and the inferences fairly deducible from them, may be clearly stated and defined. To this report should be affixed the signatures of all the professional gentlemen who witnessed the examination, and do not dissent from the conclusions. Such a procedure would be productive of great and obvious advantages. The minds of juries would not so frequently be bewildered by the discrepant or contradictory assertions of medical men; and medical evidence would acdrawn up and signed by Mr. Perkins, Mr. Ward, and myself; in which, having minutely detailed the various wounds and injuries displayed by the body of the deceased, we stated it as our " unanimous and decided opinion,"

First, *that the deceased had perished from inflammation of the brain consequent on the injuries said to have been received by him on Saturday the* 16*th instant; and that such injuries must, sooner or later, have necessarily proved fatal, notwithstanding all surgical or medical treatment which might have been employed to remedy them, and independently of any state of sickness or intoxication in which he might have been at the moment of their infliction:*

And, secondly, *that, although we could not take upon ourselves to specify precisely the implement or means by which the wounds had been inflicted, the deceased must (from circumstances to be hereafter more fully discussed) have incurred them in a struggle with some other person or persons.*

Ere we proceed to review and illustrate these positions separately and at length, it may not be improper to detail the facts which came to light respecting this mysterious business upon an inquest held on the following day.* It will be seen how far the evidence there elicited, and with every point of which I was previously unacquainted, goes to confirm the opinions delivered by myself and colleagues.

The deceased, it appears, left his house on Saturday the 16th, to go to Atherstone, with the avowed object of receiving a sum of money. He spent the whole afternoon in drinking at various public houses. Between seven and eight o'clock, somewhat " disguised in liquor," but by no means incapable of taking care of himself,† he left the town with a kinsman, on his return home. After going quire, in the opinions of the coroner and the lawyer, a degree of respectability to which it is not now always considered to be entitled, if this plan were adopted. Some hours, however, or, if possible, a night, should intervene ere such report be signed, so that there may be time for deliberate revision of it, and for the correction of any inaccuracies of opinion or expression contained in it.

* After a long and rigorous examination of the medical and other witnesses, on Friday the 29th, a verdict of *wilful murder, against some person or persons unknown,* was delivered by one of the most respectable juries before which it has ever yet been my lot to appear.

† This man declared, that he considered the deceased, while in his company, fully competent to the transaction of business.

together about half a mile, they parted, each on his respective route. Within the same distance from the spot where he was subsequently discovered, the deceased was met, in a " very jovial mood," by a carrier who knew him. They recognized each other, shook hands, and parted. From that moment all trace of him is lost. He was not again seen till found senseless and bleeding on the road, not far from his own house, which, from a comparison of dates, must have taken place within a short period from the occurrence of the injury. The ground, on which he lay, was perfectly level. With the exception of one incoherent sentence, he never spoke during his conveyance home, or for many hours after. The son, an intelligent boy, who assisted in carrying him, positively swore, that " both his breeches pockets were turned inside out:* and, during an interval in the commencement of the ensuing week, when the wounded man had, in some degree, recovered his consciousness and power of speech, he, himself, distinctly and repeatedly declared to one or two of his friends, who were questioning him, that he clearly remembered having seen, on the night of the accident, a man spring from the hedge and pass behind him; but that he felt no blow,† and was totally inconscious of all which happened from that moment. The darkness of the night was such as to prevent him from distinguishing the figure or dress of the assassin.‡

I shall now attempt to establish, by cautious inference

---

* By another witness, this important fact was, although in a somewhat hesitating tone, contradicted. But if, as, from several circumstances, I am disposed to believe, the assertion of the boy were correct, the conclusions to which it naturally leads are very decisive: either the pockets must have been turned out by the assailant of the deceased in the search for his property, and left so in the hurry and agitation of the moment; or the inversion was a *manœuvre artfully designed to give the colour of robbery to an act of violence perpetrated from other motives*. The latter is, perhaps, the most plausible conjecture. The deceased did not recieve the money at Atherstone for which he went. A few halfpence, which he had in another pocket, were not taken.

† This circumstance is, by no means, inexplicable. Violent blows on the head sometimes act upon the brain and nervous system, and extinguish sensibility with the rapidity of lightning. A friend of mine saw a highwayman approach, but never felt the blow, which severely wounded his head, and knocked him from his horse.

‡ The accuracy of the recollection of the deceased on this point can scarcely be questioned; for he, at the same time, clearly remembered, and mentioned, without any allusion being made to the subject, all the circumstances of a bargain which he had made respecting hay, on Saturday the 16th, a few hours only previous to the reception of the injury.

from facts generally acknowledged in surgical and pathological science, the positions which have been previously advanced; and to strengthen them by such arguments and illustrations, as my reading and experience most readily suggest.

Our *first* position naturally resolves itself into two parts, which demand a separate consideration.

I. *The deceased had perished from inflammation of the brain consequent on the injuries received.* This inference, I imagine, no one who reviews with unjaundiced eye, the state of inflammation displayed by the brain and its membranes, and the effusion of fluid into its ventricles—an obvious consequence of such inflammation, will be inclined to controvert. The previous healthy condition of the deceased, and the absence from the thorax and abdomen of all organic lesion, capable of explaining the sudden invasion of a formidable train of symptoms, and their fatal termination, here give a stability to our reasonings, bordering on demonstration.

Dr. Frank, of Vienna, has, I am well aware, expressed an opinion that *the red colour of the internal coat of the aorta,* which I have described as existing in this case, is the "cause of a peculiar and hitherto invariably fatal fever." But I have met with it in several of my dissections where no symptom, observed during life, had excited a suspicion of its presence. Hence, I feel little disposed to accede to the correctness of the German physician's inferences: and in such dissent I consider myself borne out, however indirectly, by the testimony of the celebrated Corvisart.* With respect to *the vascular appearance exhibited by the mucous membrane of the stomach,* it might, I think, be regarded as totally unconnected with inflammation, or other previous disease, of the gastric organ. It occupied a very limited portion of the mucous membrane; and strongly resembled the condition, of late so ably described by Dr. Yelloly, as occuring in healthy persons who have perished from the infliction of external

---

* Jamais je n'ai pu me rendre un compte satisfaisant touchant la nature et la cause de cette rougeur (de la membrane interne de l'aorte.) A-t-elle des signes et des symptômes qui lui soient propres et qui m'aient échappé?——Je l'ignore complètement. *Essai sur les maladies du cœur, &c.* Page 350. See also the very doubtful manner in which Mr. Hodgson speaks on this subject. *Treatise on the Diseases of Arteries and Veins,* page 8.

violence.* In the case of the deceased, the stomach, though necessarily implicated in the general disturbance of the system, exhibited, as far as I can learn, no sign of local or peculiar affection.

II. *The injuries, sustained by the deceased, must, sooner or later, have necessarily proved fatal, in defiance of all medical and surgical treatment, and independently of the condition of the system at the moment of their infliction.* Fracture of the basis cranii constitutes one of the most formidable accidents in surgical practice. When extensive, as in the present instance, it is, I believe, inevitably fatal. In support of this opinion, if it require any, the following considerations may be adduced: The violence of the shock which the whole mass of the cerebral organ must sustain from the operation of an external force capable of fracturing a bone so strong, and so well protected from injury both by its situation and attached muscles, as the occipital; the impracticability of determining by any diagnostic signs the precise nature of the lesion; and the utter inaccessibility of that part of the cranium, even if it were determined, to all surgical operation. Surgical writings abound with examples of the fatality of this species of accident. But I know not of any practitioner who boasts that he has detected, in the living subject, or treated with success, fracture of the basis cranii.

In our second position, we contend that *the wounds, which the body of the deceased exhibited, must have been received in a struggle with, or, at least, through the agency of, some other person or persons.* This position, though less susceptible of direct proof than the preceding one, will, we concieve, be clearly made out by reasonings drawn from the number and variety of situation which the wounds occupied. They had been alike inflicted on the *posterior* and *anterior* parts of the body—on the occiput, the loins, the forehead, the lips, and the shins. Now, had the deceased sustained his injuries in a fall *backwards*,† how are the effusion between the mem-

* See *Medico-Chirurg. Transactions*, vol. iv. page 371; and particularly the last five cases there detailed by Dr. Yelloly. In this instance, the redness both of the aortal and gastric membranes disappeared after a few hours' maceration in alcohol.

† In my evidence before the jury, I gave an opinion that such dreadful injury, as the cranium of our subject displayed, could scarcely have been inflicted on a portion of it, so strong and sheltered as the occipital bone, in a fall, "unless additional impetus and velocity had been given to the body in its descent by the application of external force." A quesbranes in *front* of the brain, the contusion of the *lips*, and abrasion of cuticle from the *shins*,* to be explained. And admitting that he had fallen *forwards*,† we are in equal difficulty with regard to the lesions of the *occiput* and *lumbar region*. But by some it may be argued, that he might have fallen in both directions. This conclusion, however, will appear destitute of probability, when we review the arguments which may be fairly brought in opposition to it: The kinsman, who accompanied the deceased out of Atherstone, declared that the latter never stumbled or fell, while in his company: to suppose that the effects of the liquor which he had drunk would be more severely felt as he proceeded, is inconsistent with daily observation and experience; and that he got no fresh supply on his road home,‡ every circumstance conspires to prove :- lastly, neither on the spot where he was discovered, nor near it, was there any precipice, any inequality of ground, or object, which could impede his progress, and thus give to his fall the appearance of a mere accident.

Let us, in brief recapitulation, connect these facts of the condition of the man when the injuries must have been received, and of the equality of the ground on

---

tion was started by some of the jury, whether, supposing that the deceased had, of himself, fallen backward and fractured the posterior part of the skull, the injury, observed on the anterior surface of the brain, might not admit of explanation, upon the same principle, as counter-fracture? To this I replied, that to discover fracture or extravasation in, or under, the part of the skull opposite to that on which the injury had been received, *while the point, actually stricken, shewed no trace of the violence*, was not uncommon in the practice of surgery; that I had myself, *in former years* met with examples of it; but that I had never seen, nor recollected to have read in surgical books, any instance in which at *both extremities* of what might be called the *line of concussion*, the effects of the violence were conspicuous. Yet even were this point conceded, the other appearances of injury in the *front* of the body remain unaccounted for.

* It was satisfactorily proved, that the deceased had no abrasion of the cuticle from his shins on the morning of the 16th of November.

† Had the deceased fallen forward, and the lesion of the occipital bone been explained on the principle of counter-fracture, to what cause shall we attribute the triangular wound of the scalp, and the contusion observed in the lumbar region? From due consideration of all the circumstances, I have little doubt that the propelling force, which knocked the deceased down, was applied above the *left eye*, and that the injuries of his occiput and loins were sustained in the violence of the fall backwards.

‡ There are very few houses on the road from Atherstone to the residence of the deceased; and at none of these does he appear to have called, on his return.

which he lay, the other circumstances brought to light on the inquest, that of his having left home for the purpose of receiving money; of the assertion of the son respecting the inverted state of the pockets of the deceased; of the figure of a man springing from the hedge, seen and recollected by the deceased himself; and, above all, that of the number and peculiar distribution and character of his wounds; and we have surely a chain of evidence sufficiently strong to warrant the medical opinions delivered on this mysterious case, and only imperfect as not yet being connected with the perpetrator of this atrocious deed.

In this protracted discussion, an importance may have been given to the preceding case, which the subject, or, at least, the mode of treating it, will not be thought to merit. However defective the latter may be, the case itself, I am convinced, affords matter for useful and interesting reflection; and even the *effort* to excite attention to a neglected branch of medical research, should command respect. Medical opinions would, doubtless, acquire the same consideration and value in the jury-room and at the bar as they do in the sphere of private practice, if they were equally consistent and generally correct. But this is not the case, and never will be till medical jurisprudence is made a distinct and essential branch of professional education, and cultivated by practitioners with the ardour which so honourably distinguishes their researches in the other departments of the science.

To stand forth as the advocate of suspected innocence, and assist in rescuing from destruction those whom prejudice or mistaken zeal would have blindly sacrificed, is an office far more grateful to the feelings, than that of exposing to public view scenes of atrocity and blood, and increasing the dreadful sum of human ferocity which the present times exhibit. But let the medical practitioner recollect that he, through whose incapacity or supineness, a crime eludes detection, is alike deficient in the duties which his own character and situation, and the interests of society, require of him. Though the deed, which we have been contemplating, still lies wrapt in mystery, the labour, employed in its investigation may not be ultimately lost. Vengeance may yet overtake the perpetrator.*

Tamworth, May, 1816.

---

* I hope shortly, to present to the notice of the readers of this Journal, the report of a trial for child-murder, with some observations upon it. Its appearance has hitherto been prevented by the pressure of more valuable matter.

